# Capturing patient experiences of care with digital technology to improve service delivery and quality of care: A scoping review

**DOI:** 10.1177/20552076241282900

**Published:** 2024-10-22

**Authors:** Patrick Dodson, Anne M. Haase, Mona Jeffreys, Caz Hales

**Affiliations:** 1 School of Health, 8491 Victoria University of Wellington, Wellington, New Zealand; 2 Health Services Research Centre, 8491 Victoria University of Wellington, Wellington, New Zealand; 3 School of Nursing, Midwifery and Health Practice, 8491 Victoria University of Wellington, Wellington, New Zealand

**Keywords:** Patient experience, digital technology, service delivery, quality of care, healthcare

## Abstract

**Objective:**

Patient experience significantly impacts healthcare quality, outcomes, resource utilisation and treatment adherence. Digital technologies offer promising approaches for capturing real-time, multi-faceted patient experiences. This scoping review investigated how digital technologies are used to capture patient experience during healthcare encounters and their potential to improve health service delivery and care.

**Methods:**

A scoping review was conducted to determine associations between patients’ use of digital technology and subsequent outcomes. Four electronic databases were searched using six combination search terms in titles and/or abstracts published between 2016 and 2022. Inclusion criteria focused on studies where patients were primary users of digital technology, reporting on their experience during care. Studies had to report on at least one outcome: health service delivery, quality of care or patient experience. Screening, data extraction and analysis were performed systematically.

**Results:**

Of the 377 studies retrieved, 20 were included. Most studies incorporated aspects allowing patients to share experiences with digital technologies. Eighty percent (*n* = 16) of studies reported improvements in patient experiences, 75% (*n* = 15) enhancements in service delivery aspects and 50% (*n* = 10) indicated improved quality of care associated with the use of digital technologies. Real-time journaling and narrative methods alongside treatment were linked to improved communication, healthcare efficiencies and patient agency. Technologies facilitating bidirectional communication were particularly associated with positive effects on patients’ sense of agency.

**Conclusion:**

Digital technologies facilitating documentation of patient experiences demonstrate potential in enhancing care quality through increased patient voice, collaboration and agency. Technologies designed to map and evaluate patients’ healthcare experiences represent a promising approach to improving healthcare outcomes, service delivery and overall patient experience. Further research is needed to establish standardised methodologies and evaluate long-term impacts across diverse populations. Integrating digital narrative medicine principles may offer valuable insights for future interventions aimed at capturing and enhancing patient experiences in healthcare.

## Introduction

The patient experience encompasses the full range of interactions, perceptions and outcomes that patients encounter throughout their healthcare journey.^1^ This concept includes subjective experiences in response to treatment such as pain levels or emotional reactions, measurable objective factors such as wait times and observations of care providers’ activities.^2^ These experiences occur at the intersection of health services and patients, serving as important indicators of healthcare quality.^3,4^ They reveal how care is experienced both interpersonally and institutionally.^5^

Improving patient experience can lead to numerous benefits, including enhanced quality of care, better health outcomes, improved equity in healthcare, more efficient resource utilisation, increased treatment adherence,^6^ and enhanced service performance.^3,7^ Recognising the potential benefits, health funders and providers are increasingly prioritising the collection of patient experience data. This information is used to assess health service performance and drive improvements in the quality of care delivered.^8^

The concept of patient experience has evolved from patient satisfaction surveys to a more comprehensive understanding of the patient's journey through the healthcare system. It includes aspects such as access to care, communication with healthcare providers, involvement in decision-making and the physical environment of care facilities.^9^ Recent research has further emphasised the importance of considering cultural competence/safety,^10^ health literacy and digital health experiences as integral components of patient experience.^11^

Digital technologies are transforming healthcare by enabling real-time data communication, treatment support and decision-making to better capture multi-faceted patient experiences as they occur.^7,12^ Various tools are being employed to gather patient data, including wearable devices like continuous glucose monitors and haptic gloves, custom-designed healthcare apps, websites and multimedia messaging systems. An emerging field within digital health is digital narrative medicine,^13^ which integrates patients’ illness narratives using digital tools such as video diaries and journalling apps, with clinical data to inform care strategies and improve service delivery. This approach aims to provide a more comprehensive understanding of the patient experience.

While digital health technologies offer many benefits, they do raise concerns about health equity.^14,15^ Access to smartphones, reliable internet and digital literacy skills are not universal and may exclude certain populations. Additionally, many digital health tools may not be designed with diverse user needs in mind, which potentially exacerbates health disparities.^16^

Despite the healthcare sector's rapid digital transformation, there is a gap in our understanding of how technology can effectively capture patient experiences throughout their healthcare journey. This information is crucial for informing health service delivery and enhancing the quality of care. Current research has primarily focused on the patient's experiences with technology-enabled care, where technology uses digital tools to support specific health conditions,^17,18^ or on exploring health information technology for patient engagement in their care within different healthcare environments.^19,20^ However, fewer studies have examined the entirety of the healthcare patient experience. This scoping review aimed to investigate whether digital methods can capture real-time patient experiences during healthcare encounters, and if these approaches can be used to develop or improve health service delivery and care. This is of particular importance in complex healthcare environments where traditional data collection methods, such as retrospective post-care interviews or surveys, may fall short in capturing the intricate details of patient experiences.

This review seeks to understand how the different aspects of patient experience, as they relate to healthcare encounters, are captured by patients when using digital technology as part of their care. Therefore, three research questions were used to guide this review:

- How are digital technologies being used to capture patient experiences?
- Does having access to digital patient healthcare experiences facilitate improvements in service delivery?
- Does digital patient healthcare experience data improve the quality of care and overall patient experience?

## Methods

### Search strategy

The scoping review methodology by Arksey and O’Malley^21^ was deemed the most appropriate approach due to its exploratory nature of uncovering how a particular population and concept within a pre-determined context has been examined within the literature.^22^ The scoping review is reported in accordance with the PRISMA-ScR reporting statement,^23,24^ except for not registering the protocol. Literature searches were performed using the CINAHL, PubMed, Embase Ovid and Scopus databases. Six search terms in combination were searched for in titles and/or abstracts published between 2016 and 2022: *digital ethnography, digital diary, patient experience, quality, delivery and healthcare*. Various synonyms for each were used to ensure the widest coverage possible in the searches. The search strategy for CINAHL is presented in the supplementary information Supplementary Table 1. This same search strategy was repeated for all the databases.

To be included in the scoping review, papers needed to be peer-reviewed and meet the inclusion criteria presented in Table 1. Importantly, patients in the studies needed to be the primary user of digital technology and the study needed to report on the patient's experience of using the technology as part of their care. The outcome measures of interest included health service delivery, quality of care and experience; a minimum of one of these outcomes needed to be reported in the study to meet inclusion criteria. All empirical research was included. Review articles were identified, and their reference lists were searched to identify additional literature not identified through the database searches.

**Table 1.** Inclusion and exclusion criteria.

*Inclusion criteria* Patient/consumer/client was the user of the digital technology Assessed patient experience Focussed on patient care/journey mapping Outcome measures reported on health service delivery improvement OR patient outcome OR patient experience
*Exclusion criteria* Health technology records, patient data without patient involvement User experience does not relate to the healthcare setting Animal studies Editorials and opinion pieces Studies exclusively involving children or their parents/caregivers Non-English language publications Published before 1 January 2016

All identified article titles and abstracts were screened according to the inclusion/exclusion criteria (Table 1) by two researchers (PD and CH) using Covidence.^25^ Any conflicts were resolved via discussion with a third researcher (MJ). Full-text articles were then examined according to the inclusion/exclusion criteria (Table 1) by two researchers (PD and CH).

### Data extraction and synthesis

Data were extracted by one researcher (PD) into a data spreadsheet within Covidence jointly developed by all researchers on the research team. Relevant data on study characteristics, methods and results were extracted. All extracted data were then checked by one of two researchers (MJ and AH) against the original articles to ensure accuracy. Identified discrepancies were resolved through discussion with all researchers. Data were analysed in a narrative synthesis, with a focus on technologies used, user acceptability, patient experience, service delivery and quality of care.

## Results

### Included studies

**Figure 1.**
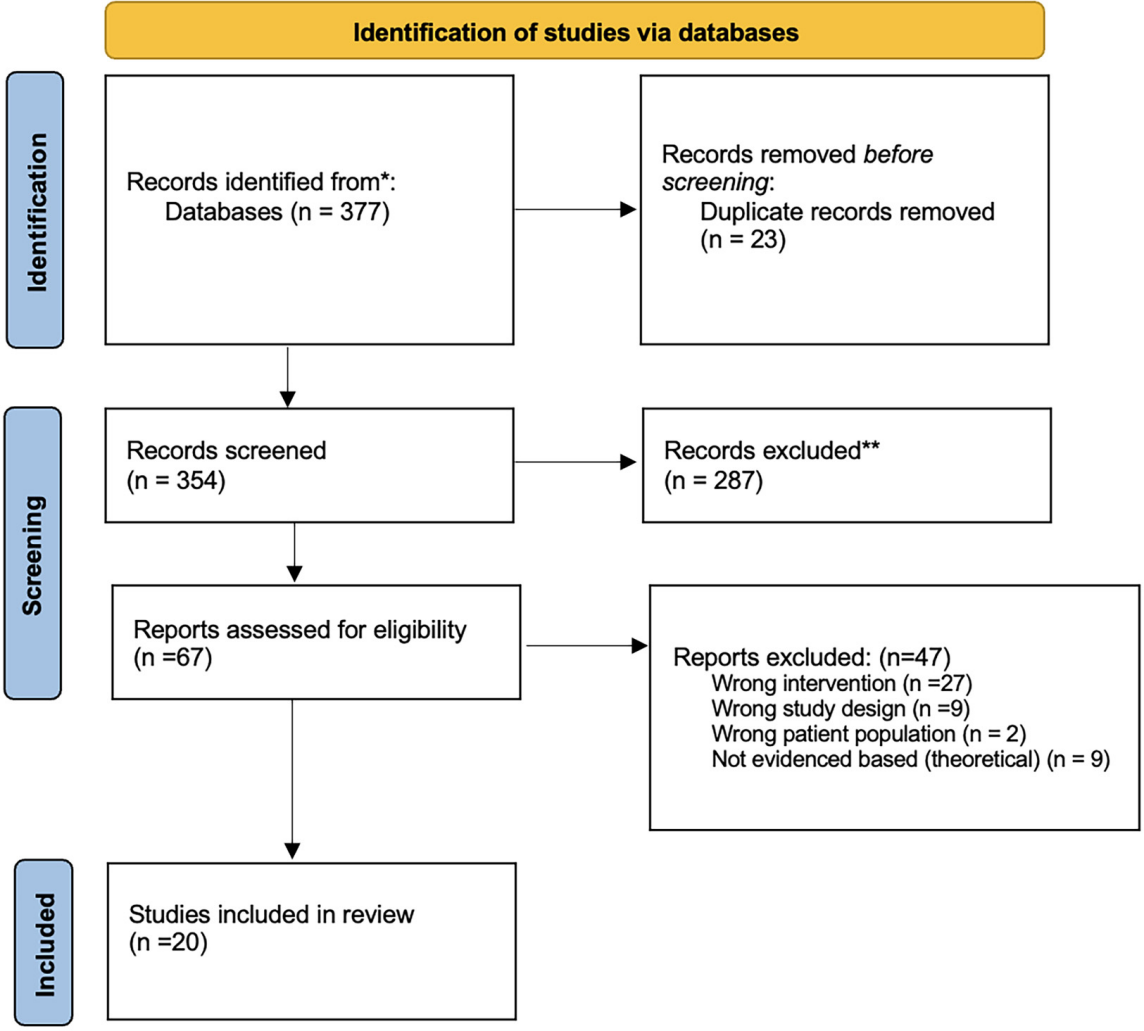
PRISMA diagram representing the scoping review literature search.

A total of 377 articles were identified: 63 from PubMed, 129 from CINAHL, 130 from Embase Ovid, seven from Scopus, 27 were added from pilot screening and 11 essential articles were added during the review process. Twenty-three duplicates were removed. Sixteen of these were identified using the Covidence duplicate detection tool^26^ and seven were manually identified as duplicates. The screening process led to 287 articles being excluded based on the inclusion/exclusion criteria, leaving 67 articles included. After examining the full text of the remaining articles, 47 articles were excluded based on the inclusion/exclusion criteria, leaving 20 articles included in the review (Figure 1). Most articles were excluded at the full-text stage because they did not report patients having direct interactions with digital technologies. Tables 2 and 3 summarises the included studies and reported outcomes.

**Table 2.** Characteristics of included studies.

Study	Study design	Study aim	Sampling and participant characteristics	Technology used	Features and use	Reported outcomes
Bohot et al. (2017), New Zealand^27^	Nonrandomised experimental study	Study aimed to explore the impact of mobile devices on clinician and patient experiences of community allied health.	Random sample 83 adult patients, 18 paediatric patients Age: Adults 54% were aged 64+ years. Paediatric 72% were aged 0–4 years Gender: Not reported Diagnosis: Broad ranging Sociodemographic: None other reported.	Smartphone with custom app.	Mobile devices were mostly used to access health information (*n* = 58) and support education/instruction (*n* = 53).	Acceptability Patient experience Service delivery Quality of care.
Cenci and Mecarelli (2020), Italy^13^	Cohort study	Pilot study aimed to assess the usefulness and feasibility of integrating narrative medicine methodologies into routine clinical practice through a digital platform.	Purposive sample 30 adult clinic patients Average age: 34 Gender: 16 men and 14 women Diagnosis: Epilepsy Sociodemographic: None other reported.	Digital Narrative Medicine Laboratory platform.	Patients accessed the platform via computer or smartphone to author his/her story guided by the prompts that may appear all together, or progressively, according to an agreed sequence.	Acceptability Patient experience Service delivery Quality of care.
Cercato et al. (2022), Italy^28^	Cohort study	Study aimed to evaluate the utility of digital narrative diaries integrated with the care pathway of patients with bone and limb soft tissue sarcomas.	Purposive sample 7 adult patients and 6 healthcare professionals Age: 37–54 years old Gender: 4 men and 3 women Diagnosis: Sarcoma Sociodemographic: None other reported.	A digital diary accessed through smartphone or desktop computer.	The DNMLAB gave the patient access to a protected personal area, called a ’diary’ (DNM), where he/she could write his/her narrative, guided by a set of narrative prompts made up by the team.	Acceptability Patient experience Service delivery Quality of care.
Coolbrandt et al. (2022), Belgium^29^	Cohort study	Study aimed to evaluate the feasibility and usability of a remote symptom monitoring system during systemic cancer treatment.	Purposive sample 111 adult patients Mean age: 59.4 SD ± xx Gender: 54 men and 57 women Diagnosis: Various cancers Sociodemographic: None other reported.	Digital online diary available via Mynexuzhealth application- secure web application.	83 (77.6%) used the mobile version, whereas 21 (19.6%) used the web version. Most of the respondents (93.5%) used the system independently of their supporters.	Acceptability Patient experience Service delivery.
Georgsson and Staggers (2017), Sweden^30^	Cross-sectional study of participants who had completed a 6-month RCT Questionnaire & semi-structured interviews following RCT.	Study aimed to understand patients’ perceptions of (a) longer-term use of the mHealth system- Care4Life, (b) specific improvements needed for Care4Life and (c) their needs for future diabetes self-management.	Random sample 10 adult patients from a larger RCT Age: 50% were 60–69 years Gender: 5 women and 5 men Diagnosis: Type 2 diabetes Sociodemographic: Ethnicity, education and profession were inquired but only education was reported. 80% were college educated.	Care4Life (Voxiva, Arlington, VA) is an interactive short messaging service (SMS) diabetes self-management system for patients.	Reviewed after 6 months of use. No other details offered.	Acceptability Patient experience Service delivery.
Jalil et al. (2019), Australia^31^	Cohort study Clinical trial Semi-structured interviews and survey within an RCT.	Study aimed to understand the patients’ user experience in telehealth, eHealth, and mHealth during a clinical trial.	Random sample 9 patients Age: 52–74 years Gender: 4 women and 5 men Diagnosis: Type 2 diabetes for minimum 12 months Sociodemographic: None others reported.	A tablet computer with an 11-in. screen, an automatic glucometer, and an automatic sphygmomanometer.	A regular patient session entailed a patient turning on the tablet and waiting to log in automatically. The patient then looked at the scheduled blood glucose and blood pressure test that was arranged by the nurse. The patient pricked a finger to get a drop of blood and put it on a strip for a blood glucose reading. The strip was then placed in the glucometer. To get the blood pressure measurement, the patient put an arm in the sphygmomanometer, which automatically took the reading.	Acceptability Patient experience Service delivery.
Jamison et al. (2018), USA^32^	Longitudinal cohort study Questionnaire at 3 and 6 months	Study aimed to determine the feasibility, tolerability, safety and efficacy of a smartphone pain application (app) among chronic pain patients over a 6-month trial.	Purposive 90 patients Age: 18–79 years Gender: 58 women and 32 men Diagnosis: Cancer and non-cancer related pain for minimum of 6 months Sociodemographic: Ethnicity (% of Caucasian) was 83.1%. Also, ‘No significant differences were found between groups on demographic variables’.	Smartphone pain app.	Patients downloaded a pain app and completed baseline questions including medical history, pain assessment and contact information. They were asked to record their progress by answering five questions every day for six months. Participants were supplied a Fitbit to track their daily activity.	Acceptability Patient experience.
Liu et al. (2022), USA^33^	Randomised controlled trial	Study aimed to explore the consumers’ perspectives on self-monitoring behaviours in mobile interventions for weight loss management and what outcome metric matter to consumers.	Random sample 48 adult participants Mean age: 38.12 Gender: 23 men and 25 women Diagnosis: Eating disorders Sociodemographic: Asian – 10%, Black – 15%, Hispanic – 17%, White – 58%, Self-identified – 2% Education and income reported with majorities being white, college educated and income over $75k USD.	Smartphone with video and text capabilities.	Participants were invited to practice self-monitoring for 3 weeks. Each week, participants were asked to submit two self-monitoring entries.	Acceptability Patient experience.
Maglalang et al. (2017), USA^34^	Cohort study Pilot RCT Semi-structured post-programme interviews after 3 months intervention and 3 months maintenance	Study aimed to assess the acceptability and cultural relevance of the PilAm Go4Health program.	Random sample 45 adult participants (22 intervention; 23 control group) Age: Mean age 58 years Gender: 28 women and 17 men Diagnosis: Non-insulin dependent type 2 diabetes Sociodemographic: Exclusively Filipino American. 56% college graduates, 69% part- or full-time employed, 84% immigrants Sociodemographic: Low-income households who qualified for Govt. subsidies in rural and urban environments in the Philippines.	(a) Fitbit, (b) Fitbit Diary App and (c) Facebook on smartphone or desktop computer.	Intervention participants were asked to (a) wear a Fitbit accelerometer daily, (b) self-report food/calorie intake and weight using the Fitbit diary app and (c) participate in the private Facebook group. Research staff posted weekly healthy lifestyle education on the private Facebook site and facilitated ad hoc virtual group discussions.	Acceptability Patient experience Quality of care.
Mendoza et al. (2021), Philippines^35^	Cohort study	Study aimed to explore opportunities and challenges in employing ‘digital diaries’ via mobile phones to track the lived experiences of people with hypertension in the Philippines.	Random and purposive samples 40 adult patients Age: 30–70 years old Gender: 26 women and 14 men Diagnosis: hypertension Sociodemographic: None others reported.	A digital platform developed by On Our Radar (OOR), a web-based application that collates participant-generated content in the form of video, photo, audio, and text captured and submitted using own mobile phones (OnOurRadar, 2015).	All participants were reminded weekly about the diary by sending a text message via the platform. The textual information submitted fell into six broad typologies: (a) biomedical information about hypertension, (b) perceptions about hypertension, (c) health concerns other than hypertension, (d) key events related to their care pathway, (e) barriers to sending diary entries and (f) requests for medical consultations.	Acceptability Patient experience Service delivery.
Minen et al. (2020), USA^36^	Cohort study	Study aimed to identify what patients view as important when tracking migraine/headaches within a headache tracking mHealth app.	Purposive sample 415 adult patients Age: Not reported Gender: Not reported Diagnosis: Migraines Sociodemographic: None others reported.	The RELAXaHEAD App for Smartphones.	In these studies, subjects were asked to input their headaches daily over a 90-day period. On average, there were 18.6 ± 39.2 notes/user, with a total of 5364 notes.	Patient experience
Moretti et al. (2021), Italy^37^	Qualitative research	Study aimed to explore the application of visual methods (photos) in the experience of people suffering from psychotic disorders.	Purposive sample 5 adult clinic patients Age: 18–35 Gender: Not reported Diagnosis: Psychosis Sociodemographic: None others reported.	Smartphone with a camera.	Patients were asked to produce photographs following 4 thematic areas (with the indication of a maximum of 5 photos per area): Fun. Time. Something indispensable. Places where I feel good.	Patient experience Service delivery Quality of care.
Neves et al. (2021), UK, Sweden, Italy, and Germany^38^	Cross-sectional study	Study aimed to evaluate patient use of virtual primary care models during COVID-19, including changes in uptake, perceived impact on the quality and safety of care and willingness of future use.	Stratified sample 6325 general population adult participants Age: 18–55+ Gender: 3241 women and 3085 men Diagnosis: Various Sociodemographic: Ethnicity data on participants was collected across three countries showing over 90% being White.	(a) Remote patient-initiated services; (b) secure messaging systems; (c) use of telephone, video and chat consultations and (d) remote triage.	During the COVID-19 pandemic, significant increases were observed for telephone consultations (+6.3%, *p* < .001), patient-initiated services (+1.5%, *n *= 98, *p* < .001), video consultations (+1.4%, *p* < .001), remote triage (+1.3, *p* < .001) and secure messaging systems (+0.9%, *p* = .019).	Acceptability Patient experience Service delivery Quality of care.
Palacios-Ceña et al. (2016), Spain^39^	Qualitative research	The study aimed to explore the experiences of patients with multiple sclerosis who performed a virtual home-exercise programme using Kinect.	Purposive sample 24 adult clinic patients Mean age: 36.69 Gender: 13 women and 11 men Diagnosis: Multiple sclerosis Sociodemographic: EDSS or Expanded Disability Status Scale reported – Score 3 EDSS was 16.4%, Score 4 EDSS was 75.5%, Score 5 EDSS was 8.1%.	XBox 360 console with Microsoft Kinect software (Kinect Sports, Joy Ride and Adventures), and a webcam.	40 sessions on the console with four sessions per week, up to 20 min per session depending on fatigue, done in participants homes. A physiotherapist monitored and supervised all interventions using online meetings via videoconferencing (webcam) to avoid adverse events.	Acceptability Patient experience Service delivery.
Schulz et al. (2010), Italy and Switzerland^40^	Cohort study	Study aimed to explore whether the intensity of a person's use of the ONESELF website was related to the perceived utility of the site for users’ ways of coping with their back pain, and with differential effects of the site on heavy and light users.	Purposive sample 748 adult general population Age: Not reported Gender: Not reported Diagnosis: chronic lower back pain Sociodemographic: None others reported.	ONESELF website	462 persons logged in to ONESELF at least once. The median number of days logged on is 11. The median total time of use among this group is 16 min and 39 s. The Library was by far the most intensely used section, with a total of 127 h. The Gym and the Forum were used for about 64 h each. All other sections were used much less intensely, at around 10 h in all cases except the Emergency room and the Homepage.	Acceptability Patient experience Service delivery Quality of care.
Taylor and Pagliari (2018), UK^41^	Case report	Study aimed to analyse the content of one cancer patient's Twitter feed in the final 6 months of her life, to determine its fit with documented end-of-life trajectories and the six dimensions of the ‘framework for a good death’.	1 adult patient Age: 29 Gender: Female Diagnosis: Sarcoma Sociodemographic: None others reported.	Twitter app	From March 2012 and, prior to her death on 23 July 2016, Kate posted approximately 12,500 tweets and attracted approximately 48,000 followers. Significant events, such as medical procedures and hospital stays generated the densest Twitter engagement. Transitions between trajectory phases were marked by changes in the relative frequency of tweet-types.	Acceptability Patient experience Quality of care.
Wall et al. (2015), Australia^42^	Mixed methods Structured questionnaires at baseline and following completion of CRT semi-structured phone interviews at 3 and 12 months.	Study aimed to explore patients’ perceptions of SwallowIT and the delivery of preventative swallowing therapy during curative radiation therapy via an asynchronous telepractice model.	Purposive sample 15 patients Age: 46–70 years Gender 15 men Diagnosis Oropharyngeal carcinoma planned for curative-intent chemo-radiotherapy Sociodemographic: ‘Most were of mid-high socioeconomic status and all but one had prior experience with technology’.	SwallowIT electronic application hosted on a secure external server and provided to patients on an ASUS Vivo SmartTab computer tablet. A Telstra USB Wi-Fi connection enabled synchronisation and upload of data to the server.	Daily therapy was completed by patients independently through SwallowIT for six weeks of CRT as per the Pharyngocise protocol. Patients also attended a 30-minute face-to-face SLP/dietetic appointment weekly, to manage side-effects, swallowing and/or nutritional issues.	Acceptability Patient experience Service delivery.
Werbaneth et al. (2020), USA^43^	Exploratory retrospective analysis	Study aimed to evaluate the feasibility of using an automated lexical analysis to identify free text notes relevant to seizure clusters.	Purposive sample 98 online diaries selected from 42,799 users Modal age: 30–39 Gender: Not reported Diagnosis: Epilepsy Sociodemographic: None others reported.	EpiDiary^TM^, a free electronic epilepsy diary. EpiDiaryTM (https://epidiary.com, Irody, Inc.).	1,096,168 total entries in the system and 247,232 free text note entries.	Patient experience Service delivery.
White et al. (2015), Australia^44^	Qualitative research	Study aimed to explore stroke survivor's experience of acceptability of having access to and use of tablets, during the first 3 months of their stroke recovery.	Purposive sample 12 adult clinic patients Median age: 73 Gender: 8 men and 4 women Diagnosis: Stroke Sociodemographic: None others reported.	iPad and Apps: Language Therapy, Angry Birds, Memory, My Mosaic, Imazing, Tangram, Fruit Ninja, Speech Sounds on Cue.	Frequency of iPad use was varied among participants. Some reported daily use characterized by ‘trying to do a little bit each day’ and ‘an hour every day’. For other participants, the use of the iPad was more variable over the loan period.	Acceptability Patient experience Service delivery Quality of care.
Yeh et al. (2011), USA^45^	Non-randomised experimental study	Study aimed to pilot a flexible telerehabilitation platform (computer game monitored through a video channel) that allows a therapist to remotely monitor the exercise regimen and progress of a patient who previously suffered from a stroke.	Purposive sample 14 adult clinic patients Age: not reported Gender: Not reported Diagnosis: Stroke Sociodemographic: None others reported.	(a) A motor rehabilitation system: vitriol games, haptic monitors and other technologies were used to interact or track progress and (b) a telecommunication system: Skype.	The three virtual rehabilitation games contained full arm and shoulder training activities with a focus on pronation and supination and hand opening and closing. Post-stroke patient volunteers with impairment of motor coordination in the upper body and staff therapists who monitored and guided the patients through virtual gameplay participated in the pre-post design experiment. The task involved completing three virtual tasks.	Acceptability Patient experience Service delivery Quality of care.

**Table 3.** Summary of key reported outcomes of included studies.

Study	Technology used	Acceptability	Patient experience	Service delivery	Quality of care
Bohot et al. (2017), New Zealand^27^	Smartphone with custom app.	Ninety-four percent (*n =* 95) of patients reported maximum levels of acceptance when a mobile device was used in their home. The remaining 6% (*n =* 6) rated acceptance as six out of seven. 93% patients (*n =* 94) reported mobile device use improved their community allied health intervention. Seven percent reported the device made no difference to their appointment.	Patients described their interventions as more responsive when the device was used to access the health record at the point of care.	Several patients acknowledged how electronic documentation eliminated some risks associated with paper documentation, such as duplication and papers being lost or misplaced.	Improved health information flow and enhanced therapeutic education and instruction were identified as key benefits of mobile device use. Patients also described a reduction in follow-up contact as an improvement.
Cenci and Mecarelli (2020), Italy^13^	Digital Narrative Medicine Laboratory platform.	Patients managed to reflect more clearly about themselves, communicating to the doctor information that otherwise would not have been taken into consideration. Of the patients involved, 75% think that the tool should be included in standard clinical practice or would like to keep on using it.	Patient opinion was positive. Both caregivers and patients agreed that the narrative digital interaction allowed them to share important aspects of treatment plans otherwise impossible to express in the traditional setting.	IDS facilitates a methodology of story interpretation devised to facilitated the comprehension and integration of an illness narrative, without applying sterile reductionism to the patient's unique and distinctive story.	The StoryMap methodology and multidisciplinary approach allowed the healthcare team to identify the priority areas of intervention for every patient to improve their experience of the illness.
Cercato et al. (2022), Italy^28^	A digital diary accessed through smartphone or desktop computer.	The average scores were generally high (>4/5) for the considered items: Diary friendliness. Diary immediacy and comprehensibility. Time management.	The highest values (≥4.7/5) regarded the possibility to express one's own point of view, the perception of effectively taking charge, and the improvement of disease awareness and self-empowerment.	These data indicate that the use of the diary did not significantly affect clinicians’ time management and the length of the scheduled visits.	According to HCPs’ evaluation of the utility items, the tool offered the opportunity to disclose relevant individual data that are otherwise not detectable (4.6/5); furthermore, it strengthened communication and relationship/alliance (4.8/5), not only between a patient and HCP but also among the members of the care team.
Coolbrandt et al. (2022), Belgium^29^	Digital online diary available via Mynexuzhealth application-secure web application.	Regarding the user-system interaction, the perceived ease of use had the highest score of all four subscales (4.7 ± 0.4), and the user control subscale scored 3.9 (±0.7). The perceived usefulness of the tool scored 3.7 (±0.9), and the perceived impact scored 4.2 (±0.7).	The mean global score (±SD) on the adapted Health-ITUES was 4.0 (±0.7) of 5. Regarding the user-system interaction, the perceived ease of use had the highest score of all four subscales (4.7 ± 0.4), and the user control subscale scored 3.9 (±0.7). The perceived usefulness of the tool scored 3.7 (±0.9), and the perceived impact scored 4.2 (±0.7). More than half of the respondents assessed the daily reminders as (very) helpful (57.2%). Only 6.5% considered them as (very) disturbing.	Almost half of the respondents (49.5%) perceived that their symptom reports were used by the nurses, but 1 out of 3 (28.4%) could not tell. Regarding the doctors’ use of the symptom reports, 31.2% (totally) agreed that their doctors had used their symptom reports, but up to 39.4% did not know.	Not reported
Georgsson and Staggers (2017), Sweden^30^	Care4Life (Voxiva, Arlington, VA) is an interactive short messaging service (SMS) diabetes self-management system for patients.	For the perceived usefulness category, 90% of patients strongly agreed and 10% agreed that the increasing Web services, mobile services, different patient support, and information services within healthcare are positive developments. The specific results for Care4Life were also positive. For the perceived use category, 60% of patients strongly agreed and 40% agreed that the Care4Life system was useful to them for monitoring and managing their disease. Seventy percent agreed they would need to visit the hospital or come in for a medical check-up less frequently, and the majority would recommend the system to others.	Most patients thought it was beneficial to track specific measurements in their day-to-day dealings with diabetes. Many of them specifically highlighted that the clearly visible measurements aided them in disease management. A group of patients experienced difficulties with having their notes about their diabetes in various places in the past. They pointed out that it was beneficial for them to be able to have everything related to their disease in one place in Care4Life.	Patients also agreed or strongly agreed that Care4Life was useful to them for monitoring and managing their disease and that the system was supportive of their discussions with their provider, and they predicted less frequent need for medical appointments when they were using the system.	Not reported
Jalil et al. (2019) Australia^31^	A tablet computer with an 11-inch screen, an automatic glucometer, and an automatic sphygmomanometer.	Patients chose to place the device in four locations: living room (*n =* 4), bedroom (*n =* 2), study room (*n =* 2) and patio (*n =* 1). Reasons mentioned were internet or phone socket availability (*n =* 3), convenience (*n =* 4), comfort (*n =* 1) and self-motivation (*n =* 1) regarding their choice of device placement.	The device motivated participants to monitor and manage their diabetes. The devices also created regimens which became a normal part of the participant’s day, fostering greater accountability and awareness. Participants also experienced frustration with the device during unresponsive or slow states.	Patients stated that they had fewer visits to the doctor during the time enrolled in the clinical trial. They indicated that they did not have to see a doctor every 3 months, which is the traditional treatment. Instead, they spoke with the nurse every 2 weeks, which decreased the doctor visits unless there was something urgent.	Not reported
Jamison et al. (2018), USA^32^	Smartphone pain app.	Of the 90 subjects in the study, 82 (91.1%) submitted daily assessment reports of pain, sleep, activity interference, mood and whether their condition had improved or worsened. Over the course of the study, 11 of the subjects asked to withdraw from the study, mostly because they said they were too busy or they did not want to start again after updating their phones. Fourteen of the subjects reported some type of technical problem with the app that restricted their daily assessments and eight did not submit any daily assessments.	Those patients who completed the six-month trial showed significant improvement in pain intensity, pain interference and pain catastrophizing compared to when they started using the app. Also, greater use of the app and frequent daily assessment entries were found to be related to an overall improvement in mood. Contrary to the hypotheses, frequent use of the app did not have a positive effect on pain or activity. Also, those who were most satisfied with the app, in general, were less active and reported greater disability than those who were least satisfied with the app.	Not reported	Not reported
Liu et al. (2022), USA^33^	Smartphone with video and text capabilities.	Regarding overall satisfaction, there was no significant difference in satisfaction scores between groups (*U* = 232.5, *p = *.26), based on the seven-item Satisfaction subscale of the Usefulness, Satisfaction, and Ease of Use (USE) Questionnaire.	The results suggest it may be useful for binge eating and weight management interventions to engage consumers in identifying and monitoring additional metrics beyond binge eating and weight that matter to them.	Not reported	Not reported
Maglalang et al. (2017), USA^34^	(a) Fitbit, (b) Fitbit Diary App and (c) Facebook on smartphone or desktop computer.	Over half (*n =* 26, 57.8%) of the respondents stated that the culturally tailored support in terms of materials and staff enhanced their engagement.	Several respondents discussed how the intervention helped them change from a negative state of mind about their diabetes management to taking a more proactive stance. Many felt they could not make the necessary changes on their own.	Not reported	Increased engagement, retention, and positive behaviour change among participants can be attributed to the utilization of research staff who share cultural traditions and beliefs with the participants.
Mendoza et al. (2021), Philippines^35^	A digital platform developed by On Our Radar (OOR), a web-based application that collates participant-generated content in the form of video, photo, audio, and text captured and submitted using own mobile phones (OnOurRadar, 2015).	While all participants initially engaged with the platform, by the end of the study only six could be considered to have actively engaged throughout, wherein they sent diary entries more than once a week, two of whom posted entries daily even without probing from the researchers. Eleven participants were semi-active, sending entries once a week or from time to time, especially when they had just received their weekly mobile credit. Twenty-three were infrequent users, who engaged with the platform irregularly.	Some participants needed the help of younger family members to submit entries. There were also instances when entries were submitted after changing their mobile phone number or using a borrowed phone from a relative or a neighbour.	The instantaneous exchange offered by mobile devices allowed us to immediately monitor data quality, ask for elaborations and follow-up on developing issues.	Not reported
Minen et al. (2020), USA^36^	The RELAXaHEAD App for Smartphones.	Not reported	Having the flexibility to provide these widely varying pieces of relevant information in a free-text mHealth app was considered critical for people with migraine to specialize their own personal management and for physicians to determine appropriate and effective treatments.	Not reported	Not reported
Moretti et al. (2021), Italy^37^	Smartphone with a camera	Not reported	The data collected through the interviews showed that the proposed experience (joint use of two visual techniques) had been considered positive by both users and operators. According to many users, images managed to ‘focus’ on aspects that were difficult to convey and ensured timely processing of content.	The healthcare professionals involved also confirm the potential of this tool which, when combined with the traditional interview, can deepen the patient's knowledge by overcoming the verbal barriers that often make it difficult to reconstruct the individual experience of illness.	In accordance with other studies, our research suggested that the use of methods that involved patients and healthcare professionals could be helpful to evaluate and improve healthcare.
Neves et al. (2021), UK, Sweden, Italy and Germany^38^	(a) Remote patient-initiated services; (b) secure messaging systems; (c) use of telephone, video and chat consultations and (d) remote triage.	Willingness to use telephone consultations increased with increasing age; for video, chat consultations and online triage, interest in future use reached a peak at 35–54 years, progressively declining in older groups (all *p < *.001). Not wanting to use any of these remote care solutions was higher in men (14.7 vs. 12.6, *p = *.017), those with higher digital literacy (24.4 vs. 12.0, *p < *.001), and those in higher age bands (*p < *.001). Interestingly, participants with higher eHEALS score consistently reported a lower intention to use each one of the remote care technologies evaluated (all *p < *.001).	Of the 3976 (62.9%) responses to the experience item, the majority reported having had either a good or very good experience (59.8%, *n =* 2379). Only a small minority reported a bad or very bad experience (8.4%, *n =* 333). Lower rated experience was observed in women (2.4 ± 1.0 vs. 2.3 ± 0.9, *p < *.001) and those with low literacy respondents (eHEALS ≥ 26) (2.3 ± 0.9 vs. 2.8 ± 1.0, *p < *.001).	Virtual care technologies were most often reported as having positively impacted on timeliness (60.2%, *n =* 2793) and efficiency (‘avoiding waste’) (55.7%, *n =* 2401) of care, followed by effectiveness (46.5%, *n =* 1802), safety (‘avoiding harm’) (45.5%, *n =* 1822) and patient-centredness (45.2%, *n =* 45.2). Equity was least often reported as having been positively impacted (42.9%, *n =* 1726).	Globally, digital patient-initiated services were most often reported as having a positive impact on the quality of care received across respondents (61.0%, *n =* 2171), followed by telephone consultations (56.5%, *n =* 2102), and video consultations (44.1%, *n =* 1246). Online triage (42.5%, *n =* 1294), chat consultations (40.3%, *n =* 1177) and remote monitoring (36.6%, *n =* 968) were least often reported as impacting positively on the quality of care.
Palacios-Ceña et al. (2016), Spain^39^	XBox 360 console with Microsoft Kinect software (Kinect Sports, Joy Ride and Adventures), and a webcam.	87.4% of the sample said they were highly satisfied with the intervention.	Results showed that patients with MS regained the feeling that they were in control over their bodies and their lives thanks to the performance of games using Kinect.	Participants viewed the intervention as positive in that they did not have to travel to the hospital for treatment. For some, this intervention was the only form of treatment.	Not reported
Schulz et al. (2010), Italy and Switzerland^40^	ONESELF website	25% reported that ONESELF contributed to increasing their knowledge about back pain. An additional 58% said ONESELF had contributed sufficiently to knowledge.	There was no indication that heavy users of ONESELF reported more benefits than medium or light users. However, the interviewed sample considered ONESELF especially useful to build an individualized understanding of their situation: The ONESELF intervention helped them to construct their personal frame of reference about the nature and the course of their cLBP.	Users also acknowledged in majority ONESELF's contribution to managing their back pain: 12% said the site had contributed significantly, and 57% said it had contributed sufficiently to managing pain.	The next most frequently acknowledged benefits were improvement of communication with doctors (56%) and family and colleagues (55%).
Taylor and Pagliari (2018), UK^41^	Twitter app	Through her experiences as a patient, she and her husband founded the ‘#hellomynameis’ campaign encouraging healthcare staff to introduce themselves to patients.	Kate Granger had many online followers, who helped to lift her spirits during periods of difficult treatment and distress, and studying the patterns of support and reciprocity in these digital spaces may suggest new ways in which to help patients nearing death.	Not reported	While the analysis was at the structured end of the digital ethnographic spectrum, it nevertheless shows the value of such methods for understanding how terminal disease is experienced by and affects individuals, how they cope, how support is sought and obtained and how patients feel about the ability of palliative care services to meet their needs at various stages.
Wall et al. (2015), Australia^42^	SwallowIT electronic application hosted on a secure external server and provided to patients on an ASUS Vivo SmartTab computer tablet. A Telstra USB Wi-Fi connection enabled synchronisation and upload of data to the server.	At baseline, attitudes towards SwallowIT were positive. Most agreed/strongly agreed that they felt comfortable, confident, motivated, and supported to complete their therapy via SwallowIT.	Participants found SwallowIT intuitive and easy to use. Participants experienced a range of extrinsic and intrinsic motivators for completing therapy using SwallowIT. Most reported that SwallowIT itself motivated them.	Participants also commented on the flexibility and convenience of the asynchronous design for completing therapy around other life commitments, with some stating that this flexibility was a key factor in their preference for SwallowIT over face-to-face therapy. Participants also discussed a range of factors which made completing their exercises during CRT difficult such as CRT-induced toxicities and feelings of apathy during treatment which hindered motivation.	Not reported
Werbaneth et al. (2020), USA^43^	EpiDiaryTM, a free electronic epilepsy diary. EpiDiaryTM (https://epidiary.com, Irody, Inc.).	Not reported	Diary FTN contain information of high importance to people with epilepsy, written in their own words.	Improved patient and family understanding of the onset of a cluster could lead to more careful use of rescue medications to reduce unnecessary healthcare resource utilization.	Not reported
White et al. (2015), Australia^44^	iPad and Apps: Language Therapy, Angry Birds, Memory, My Mosaic, Imazing, Tangram, Fruit Ninja, Speech Sounds on Cue.	Overall access to an iPad within the first few months’ post-stroke promoted confidence, access to health information, independence, and socialization. Some participants described the challenges of learning to use technology that was new to them. Feelings of being challenged towards use of an iPad contributed towards many participants feeling hesitant or ‘daunted’. However, despite feelings of apprehension participants persevered with exploring the use of iPads as part of their opportunity for recovery.	Participants with significant cognitive or language impairments reported difficulties in setting up or following instructions. The most reported ongoing difficulties pertained to not being able to respond to App prompts/requests quickly enough. However, in all circumstances, participants were able to solve any given problem (e.g., logon, program feedback or instructions) independently, with the assistance of family or advice from their treating therapist during a home visit or video conferencing session.	Participants felt empowered by having an iPad as it mediated the experience of organizational constraints, such lack to access therapists, by enabling them to do more therapy and subsequently make gains towards their recovery.	In addition, participants perceived that having access to having an iPad during their rehabilitation contributed to improved outcomes. For example, three participants reported that regular use of an iPad, with focused use of their hemiplegic arm, promoted functional improvements.
Yeh et al. (2011), USA^45^	(a) A motor rehabilitation system: vitriol games, haptic monitors and other technologies were used to interact or track progress and (b) A telecommunication system: Skype.	An active mood before the experiment was positively correlated with patients’ willingness to persist in the therapy after the game, *r*(14) = .699, *p < *.005, *N =* 14. The results suggest that if patients report that they feel active and motivated, they tend to state that they are willing to continue in the therapy after the gameplay regardless of their experience with the telerehabilitation games.	The assessment of psychosocial variables showed that negative feelings were significantly lessened after the gameplay. Overall, we found that participants felt less confused, less depressed, and more relaxed after playing the games, supporting hypotheses.	When therapists felt more immersed in the telerehabilitation experience and fully engaged with the presence of the patients, patients’ general sense of acceptance of the remote rehabilitation session and positive evaluation of the overall experience were higher.	The result showed that the correlation of the participant's overall satisfaction and the therapists’ feeling of presence was statistically significant, *r*(8) = .770, *p < *.05, *N =* 8. Overall, therapists whose patients rated the telerehabilitation system more useful and satisfactory were more likely to feel higher co-presence during the gameplay and vice versa, supporting H2.

### Technologies used

Studies in this review employed a range of hardware and software technologies to deliver treatments,^31,33,34,38,39,42,45^ manage care^29,30,36,44,46^ and to gather feedback from patients.^13,28,30,34–36,40,43,46^ Many of these studies used standard hardware which patients already owned including smartphones, tablets and desktop computers.^13,28,30,34,36,37,46,47^ Other studies provided a Fitbit,^34,46^ X-Box^46^ or treatment-specific technologies such as haptic gloves^27^ or continuous glucose monitors (CGM).^31^

Sixteen studies (80%) created custom-designed software solutions to deliver one or more patient interactions: four (20%) to deliver online training,^27,34,39,42^ eight (40%) to monitor patient progress,^30,31,33,34,36,39,43,46^ two (10%) to share patient records^27,29^ or seven (35%) to receive patient feedback.^13,28,35,37,40,42,46^ These applications were either written for iOS or Android systems or were web-based and accessible through the patient's or clinician's web browser. Two exceptions to this were the use of X-Box gaming software^39^ and a custom desktop application designed specifically to deliver robotics-based physical therapy.^45^

The hardware and software used in these studies focused on delivering disease-specific information such as how to manage diabetes^30,31,34^ or back pain.^40^ Information was offered through a range of formats from synchronous online clinical consultations with live human health providers to asynchronous care guidelines posted to web pages or tablet-based apps. No studies mentioned the use of artificial intelligence as a component of their technology system. Patients typically interacted with this information by accessing the data through the chosen hardware/software solution and were often tracked to monitor progress, behaviours or significant changes. Some studies delivered diabetic care guidelines to patients while tracking their responses via a CGM device and or a sphygmomanometer.^31^ Furthermore, patients in these studies also provided personal feedback via SMS, offering another level of information to clinicians.^30,35,43^

While most of the studies focused on delivering treatments or managing care, three studies used hardware and software solutions specifically to gather patient experiences. For instance, Coolbrandt et al.^29^ used digital patient diaries to track patient experiences where self-reported treatment-related symptoms acted as prompts for health professionals to respond with advice for self-management. Two other studies^13,28^ used digital technologies such as video, audio and text capture to gather patient narratives with specific prompts for (a) biomedical information about hypertension, (b) perceptions about hypertension, (c) health concerns other than hypertension, (d) key events related to their care pathway, (e) barriers to sending diary entries and (f) requests for medical consultations. In this way, smartphone technology was employed to facilitate what the authors noted as Digital Narrative Medicine, a clinical practice which invites patients to share rich feedback using technology.^13,28^

### Acceptability of using digital technology

Seventeen studies (85%) reviewed reported some measure of acceptability among patients using the technology as part of their care or illness/disease management.^13,27–31,33,35,38–40,42,44–48^ Of those studies (*n* = 17) that reported acceptability, only three (15%) identified that patients experienced technical challenges, problems or the need for technical assistance,^33,44,46^ while the rest reported positive experiences. For instance, six (30%) of the 17 studies mentioned that the technology intervention improved interactions with their providers;^13,27,34,38,42,45^ five (25%) of studies highlighted improved patient understanding regarding their condition^13,30,34,40,44^ and four (20%) of the studies mentioned that patients found the technology useful, convenient or friendly.^28–31^.

Patients and researchers further reported more nuanced aspects of technology acceptability. This included the need for ongoing training or support,^33^ the recognition that one type of technology intervention did not suit all patients,^35,36^ that different countries or cultures exhibited different levels of acceptability based on digital literacy, conservative values or government regulations^38^ and that for some, a financial component may be important in terms of money to be invested or saved,^39^ or that acceptability can improve over time with use.^44^

### Patient experience

All the studies (*N* = 20) in this review focused on the subjective component of patient experience where the intention of the technology was to support treatment regimens. None of the studies specifically focused on objective factors of care interactions, such as waiting times, or observations of provider care activities. Fourteen studies (75%) in this review cited positive patient experiences.^13,27–30,37–40,42,45–48^ Patients shared that they had more responsive interactions with carers/clinicians, allowing for improved communication both ways.^13,27,28,30,36,40^ Patients also reported positive treatment effects, noting that the technologies made monitoring symptoms,^30,40^ creating care regimes^31,33^ or following instructions more manageable.^42^ Additionally, patients experienced improvements in symptoms where the technology helped mitigate pain intensity,^40,46^ decreased fatigue^45^ or loneliness.^47^ Positive patient outcomes reported in the studies included improved patient mood, agency and proactivity;^34,36,45^ a sense of taking charge or control;^27,39^ a sense of organisation,^30^ being more accountable and aware,^31,42^ less catastrophising,^46^ feeling empowered,^34^ increased focus,^37^ and developing a sense of community.^47^ However, two studies (10%) noted that patients experienced the need for family assistance,^35,44^ or that they experienced difficulties interacting with the technology due to their disease or condition.^44^

Five studies (25%) used digital technologies as an ethnographic resource, providing a pathway for patients to share narrative details of their health journey that may have otherwise not been part of a clinical conversation.^13,28,34,37,47^ The technology allowed for information to be provided ‘in the moment’ and in the context of their lives and current health condition. This facilitated the opportunity for patients to share or gain support from other patients or to help other stakeholders and family members better understand their condition.

### Service delivery

Fourteen of the studies (75%) reviewed noted that digital technology improved some aspect of service delivery.^13,27–31,35,37–40,42–45^ Of these, eight (40%) reported improved levels of communication between patients and their health professionals and carers.^13,29–31,37,45^ Technology facilitated a more comprehensive understanding of the illness narrative,^13^ digital interactions reduced the need for medical appointments,^30,31,39^ digital communication increased the speed of data delivery and monitoring^35^ and improved the clarity of clinical information.^37^ Other delivery improvements included the elimination of some risks associated with paper documentation;^27^ improved overall efficiency, effectiveness and safety;^38^ improving patient’s (and family) understanding of complex symptoms and therefore reducing healthcare resource utilisation^43^ and the ability to focus on patient experiences through mutual virtual engagements.^45^

### Quality of care

Ten of the studies (50%)^13,27,28,34,37,38,40,44,45,47^ reviewed discussed how digital technology improved the quality of care for patients at various levels and interactions in service delivery. Examples of quality improvement pathways included improved information sharing between clinician and patient^27^ and the identification of priority interventions through story mapping.^28^ These interventions identified relevant patient data which were not detectable through standard interactions.^28^ In other studies, the technology itself improved therapeutic outcomes^44^ or provided the ability to evaluate or improve healthcare quality through observing detailed patient experiences.^37,47^

## Discussion

This scoping review sought to investigate the utilisation of digital technology by patients to document their care episodes during healthcare interactions, with the aim of enhancing patient experience, health service delivery and quality of healthcare. However, our review revealed a paucity of literature pertaining to the deliberate application of digital methods for capturing patients’ healthcare experiences. Among the 20 studies analysed, only two^13,28^ explicitly captured patient care experiences. Nevertheless, the remaining studies incorporated design elements that facilitated patients to share their healthcare experiences through digital technologies. These experiences, although primarily centred around subjective patient perceptions of treatment, were communicated via various digital mediums including text messaging, video conferencing and clinical forums. Despite this limitation, the findings indicated numerous positive outcomes in terms of service delivery improvements, enhanced quality of care and overall patient experience.

The utilisation of digital technology for chronic health management was predominantly perceived as acceptable by patients across multiple studies.^13,27–31,33,35,38–40,42,44–48^ However, the precise nature of this acceptability, whether it pertained to the technology's usability, disease management efficacy or overall healthcare experience, remained ambiguous. This stemmed from a lack of comprehensive reporting on the acceptability frameworks utilised in the analysis of the technology's user experience component. The absence of such methodological clarity impedes a nuanced understanding of the factors driving patient acceptance of these digital interventions in healthcare settings.

Digital technology has emerged as a novel and acceptable medium for patient self-reported health data.^13,28,34,37,47^ Moreover, digital technologies and devices were found to provide a new modality for disease management communication that was not previously available.^13,27,28,30,36,40^ In instances where technology facilitated bidirectional communication between patients and healthcare professionals regarding health status or care episodes, it enhanced clarity, fidelity, accuracy and accessibility of clinical data. These communications enabled patients to share experiential feedback, often augmenting healthcare professionals’ contextual understanding of the condition or patient experience. The development of such technology allows healthcare professionals to tailor treatment responses and patient education, resulting in individualised care plans that have the potential to improve patient management and health outcomes.^49^

For a subset of patients, digital technology was the only modality of care delivery. Given recent COVID-19 self-isolation requirements, the enhancement of communication through augmented patient interactions via digital technology warrants consideration as an important approach for healthcare provision. This consideration is particularly important given that digital interactions have been shown to increase engagement, retention and positive behaviour changes among patients across a broad spectrum of electronic health systems.^50,51^

With respect to the management of specific health conditions, some patients reported that the digital intervention facilitated improved pain management^40,46^ or improved motivation in adhering to treatment regimens.^34,36,45^ B extending the communication both ways, those studies that shared patient records or reduced the inaccuracies of paper-based communications also had a positive effect on patient collaboration. Consequently, this enhanced collaboration fostered a sense of agency for patients.^34,36,45^

Digital technology has been observed to facilitate an enhanced sense of autonomy, voice and agency for patients, while simultaneously providing more appropriate treatment opportunities and improving upon outdated service delivery. These findings align with a systematic review of patient experiences of using technology-enabled care, which reported an evolving sense of independence and empowerment about their condition, greater autonomy and increased feelings of security.^17^ Additionally, our review identified that digital technology advancements have effectively reduced the temporal gap between patient and healthcare professional interactions, optimised data flow and created opportunities for instantaneous responses. This has engendered a perception among patients of continuous care provision. These experiences and attitudes were frequently communicated either through the technology intervention itself or during traditional clinical consultations. This type of feedback is an important aspect in Digital Narrative Medicine.^13,28^

Digital Narrative Medicine may be used to enhance patient experience, healthcare delivery and hence, improve patient outcomes.^13,28^ This technique conceptualises the clinical experience as a narrative and exemplifies how digital technologies can enable a clinical interpretation of the described narrative.^13^ While only two studies^13,28^ in our review explicitly aimed to apply Digital Narrative Medicine principles, all studies utilised digital technology to capture clinical data in some form, which can be used to enhance patient experience. Given that positive patient experience has been shown to improve quality of care, health outcomes and equity,^3,6,7^ the implementation of digital technologies specifically designed to capture and enhance patient experience may add the essential narrative element. This narrative component could serve as the link for improved resource utilisation and treatment adherence if narrative data can be successfully integrated into clinical practice.

Finally, in terms of patient integration, it was difficult to assess health equity or disparities in the use of digital health technologies as only seven (35%) of the articles reported more broadly on sociodemographic characteristics such as ethnicity, education or employment status.^30,32–34,38,39,42^ As these studies spanned across several countries and focused primarily on identifying clinical characteristics, no clear themes emerged as to population disparities. More research is needed to assess issues in access to and implementation of digital health technologies within diverse and larger populations.

### Strengths and limitations

The use of standard scoping review methodology means we are confident that the breadth of articles included reflects the range of contexts in which digital technologies are being used to capture patient care episodes and improve upon patient experiences. However, our review should be interpreted with some limitations in mind. A key concern is that any digital intervention will not achieve equal uptake across patient groups, and therefore, if beneficial, may potentially widen existing health inequities.^14^ This may occur due to the known digital divide, for example, in Aotearoa, New Zealand, the lack of internet access has been found to be lower for Māori, Pacific Peoples, those living in rural areas, and older people are all likely to have lower levels of internet access.^52^ Nevertheless, these are groups for whom the health system remains less accessible than others.^53^ A recent call for the development of best practice guidelines for the implementation of digital health innovations, to ensure that these interventions are pro-equity,^14^ is timely and essential.

This scoping review included studies employing a wide array of software and hardware solutions aimed at enhancing care delivery, facilitating clinician–patient communication and understanding the patient experience. However, a notable limitation was the absence of standardised protocols or applications in these technological interventions. Furthermore, there is a need for investigation into the potential marginalisation of certain populations in accessing healthcare due to technological disparities. This digital divide warrants careful consideration to ensure equitable healthcare provision in an increasingly digitalised landscape. Finally, a robust discussion regarding the ownership of data and data sovereignty is required before mainstream adoption of certain technologies.

As the use of digital technology varied across the range of the studies reviewed, so did the discussion of various resources applied, adherence to digital strategies, process indicators and efficiencies gained. This variance makes it impossible to form any overarching conclusions around the efficacy of digital technologies applied to capturing patient's healthcare experiences. However, there are enough promising results to consider developing new frameworks or standards of practice, which intentionally apply Digital Narrative Medicine and patient experience informed data to clinical practice and health service delivery.

One limitation of the digital technologies reported in this scoping review was the lack of information regarding design fidelity, and specifically, patient and public involvement in the development of these technologies. Only one study provided a comprehensive discussion on the development pathway to Digital Narrative Medicine.^54^ While it is acknowledged that such developmental processes are often reported separately, studies focusing on outcome data, such as those included in this review, would benefit from an explicit acknowledgement statement around design fidelity.

It is important to note that quality assessments are not standard practice in scoping reviews. Consequently, the results should be interpreted with caution as the included papers were not necessarily of high methodological quality. Finally, due to time constraints, the scoping review protocol was not pre-registered. However, it should be noted that no significant deviations from the protocol were made during the course of the review.

### Practice implications

The findings of this review have several implications for the design, development and implementation of digital technologies, as well as for the measurement of patient experience outcomes via these technologies. A key design feature identified as important to patients was the capacity for real-time journalling or documentation of subjective experiences of care. This feature aligns with the principles of patient-centred care and has the potential to provide rich, contextual data that can inform healthcare delivery and quality improvement initiatives.

A notable gap in the existing literature is the measurement of patient experiences that encompasses objective factors and observations of care practices. This gap represents an area for future research, specifically in developing comprehensive methodologies that integrate both subjective and objective measures of patient experience.

Importantly, digital technologies designed for one population group may not be generalisable or acceptable to others. It is, therefore, critical that any potential digital intervention is co-designed with next and end users,^55^ and carefully evaluated to ensure that potential intervention-generated inequities are identified and rectified through adaptation of the design of the intervention. Such participatory approaches can enhance the relevance, acceptability and effectiveness of digital interventions across diverse patient populations.

## Conclusion

This scoping review reveals a paucity of literature examining the utilisation of digital technology for capturing patient experiences throughout their healthcare journey. Digital technology has the potential to facilitate critical and timely patient feedback, which not only clarifies treatment priorities, but also empowers patients to engage in their care with enhanced agency, a process known to have inherent therapeutic value. Digital technology that incorporates patient experiences and narratives demonstrates promise in improving the quality of care by enabling patient voice, fostering collaboration between patients and healthcare providers and enhancing patient agency. The development and implementation of digital technologies specifically designed to evaluate patient experiences in the healthcare setting is one approach to address health outcomes, quality of care and improved service delivery. However, while these technologies offer significant potential, their efficacy and impact require further rigorous investigation.

## Supplemental Material

sj-docx-1-dhj-10.1177_20552076241282900 - Supplemental material for Capturing patient experiences of care with digital technology to improve service delivery and quality of care: A scoping review Supplemental material, sj-docx-1-dhj-10.1177_20552076241282900 for Capturing patient experiences of care with digital technology to improve service delivery and quality of care: A scoping review by Patrick Dodson, Anne M. Haase, Mona Jeffreys and Caz Hales in DIGITAL HEALTH

sj-pdf-2-dhj-10.1177_20552076241282900 - Supplemental material for Capturing patient experiences of care with digital technology to improve service delivery and quality of care: A scoping review Supplemental material, sj-pdf-2-dhj-10.1177_20552076241282900 for Capturing patient experiences of care with digital technology to improve service delivery and quality of care: A scoping review by Patrick Dodson, Anne M. Haase, Mona Jeffreys and Caz Hales in DIGITAL HEALTH
