# Supplementary material for: Capturing patient experiences of care with digital technology to improve service delivery and quality of care: A scoping review
Source: Digit Health. 2024 Oct 22;10:20552076241282900. doi: 10.1177/20552076241282900 (PMC11500239; doi:10.1177/20552076241282900)
Supplement: sj-docx-1-dhj-10.1177_20552076241282900 - Supplemental material for Capturing patient experiences of care with digital technology to improve service delivery and quality of care: A scoping review [file sj-docx-1-dhj-10.1177_20552076241282900.docx]

**Supplementary Information**

**Supplementary Table 1-** CINAHL Search Terms (Literature search terms performed 10 November 2022)

| Search completed Oct 11, 2022    ("digital ethnography" OR "digital diary") AND "patient experience" AND (quality OR delivery) AND healthcare  <https://search-ebscohost-com.helicon.vuw.ac.nz/login.aspx?direct=true&AuthType=sso&db=ccm&bquery=(%26quot%3bdigital+ethnography%26quot%3b+OR+%26quot%3bdigital+diary%26quot%3b)+AND+%26quot%3bpatient+experience%26quot%3b+AND+(quality+OR+delivery)+AND+healthcare&cli0=FT&clv0=Y&cli1=AA1&clv1=Y&cli2=DT1&clv2=201701-202312&type=1&searchMode=Relevancy&site=ehost-live&ssl=y&custid=s5491658> | ("digital ethnography" OR "digital diary") AND "patient experience" AND (quality OR delivery) AND healthcare | **Limiters** - Full Text; Abstract Available; Published Date: 20170101-20231231  **Expanders** - Apply equivalent subjects  **Narrow by Subject Geographic:** - Canada  **Narrow by Subject Geographic:** - Australia & New Zealand  **Narrow by Subject Geographic:** - USA  **Narrow by Subject Geographic:** - UK & Ireland  **Narrow by Language:** - English  **Search modes** - SmartText Searching |
| --- | --- | --- |
